# Deep Learning-Based Classification for Melanoma Detection Using XceptionNet

**DOI:** 10.1155/2022/2196096

**Published:** 2022-03-22

**Authors:** Xinrong Lu, Y. A. Firoozeh Abolhasani Zadeh

**Affiliations:** ^1^Gannan University of Science & Technology, Ganzhou 341000, Jiangxi, China; ^2^Department of Surgery, Faculty of Medicine, Kerman University of Medical Sciences, Kerman, Iran

## Abstract

Skin cancer is one of the most common types of cancer in the world, accounting for at least 40% of all cancers. Melanoma is considered as the 19th most commonly occurring cancer among the other cancers in the human society, such that about 300,000 new cases were found in 2018. While cancer diagnosis is based on interventional methods such as surgery, radiotherapy, and chemotherapy, studies show that the use of new computer technologies such as image processing mechanisms in processes related to early diagnosis of this cancer can help the physicians heal this cancer. This paper proposes an automatic method for diagnosis of skin cancer from dermoscopy images. The proposed model is based on an improved XceptionNet, which utilized swish activation function and depthwise separable convolutions. This system shows an improvement in the classification accuracy of the network compared to the original Xception and other dome architectures. Simulations of the proposed method are compared with some other related skin cancer diagnosis state-of-the-art solutions, and the results show that the suggested method achieves higher accuracy compared to the other comparative methods.

## 1. Introduction

The most common cancer in the United States is skin cancer, which occurs in the tissues of the largest part of the body, i.e., the skin [1]. The skin blocks heat, sunlight, wounds, and infections [2]. The skin has three layers: *epidermis*, dermis, and hypodermis [3]. The *epidermis* is the outmost layer of skin, which creates the skin tone and makes a waterproof barrier for the skin. The dermis is the second layer that comprises rough connective tissue, sweat glands, and hair follicles. And finally, the hypodermis, as the lowest layer, has been made by connective tissue and fat [4]. The main threat to skin is skin cancer. Skin cancer (like melanoma) is one of the most common types of cancer in the world, accounting for at least 40% of all cancers. It has been predicted that about 9,500 people in the US are diagnosed with skin cancer every day [5].

While cancer diagnosis is based on interventional methods such as surgery, radiotherapy, and chemotherapy, studies show that the use of new computer technologies such as image processing mechanisms in processes related to the diagnosis and classification of cancers has been acted successfully [6]. Among different kinds of skin cancers, melanoma is known as the 19th most commonly occurring cancer in men and women [7]. In 2018, about 300,000 new cases were recognized [8]. Based on the Cancer Cell Organization, melanoma cancer with 15000 cases is the fourth most common cancer in the world [9]. Also, based on this organization, melanoma is the 9^th^ most common reason for cancer death in 2019 [10]. Skin cancer diagnosis is known as a tough task because of the advent of diverse kinds of skin lesions, especially melanoma and carcinoma [11]. Several noninvasive methods have been proposed to avoid unnecessary biopsy for diagnosing melanoma [12]. Most of the methods usually contain three main parts: segmentation, features extraction, and classification [13]. Several works were done in this case [1, 14–16]. Bansal et al. [17] proposed a technique for melanoma diagnosis based on deep learning-based image feature extraction. The authors used convolutional neural networks (CNN) for the extraction of the features based on the transfer learning and some different classifiers including k-nearest neighbor (KNN), AdaBoost, and random forest (RF) to the final classification. The method was performed to the ISIC dataset, and the results showed the accuracy of each classifier. The method was a good technique, but due to the complex configuration, it needs more time for doing the process.

Xu et al. [6] presented a method for early detection of melanoma. They used a sequential methodology including image noise reduction, image segmentation, feature extraction, and classification. The method of segmentation in the study was based on an optimized convolutional neural network (CNN) using the satin bowerbird optimization (SBO). To extract just important features from the segmented images, SBO was utilized. At last, Support Vector Machine (SVM) was used to classify the images based on the achieved features. The method was performed to American Cancer Society database, and the results showed efficient results for the proposed method. However, the method provided good results, using the proposed method, and due to the combination of deep learning and the SBO algorithm, it provided complex system.

Razmjooy et al. [18] proposed a diagnosis technique for determining the skin malignant cancer. They first eliminated extra scales by the smoothing and edge detection. Then, the method segmented the region of interest. The additional information was removed by mathematical morphology. The model used an optimized MLP neural Networks (ANN) based on World Cup Optimization algorithm to get more efficient results. In that study, the authors used the optimized ANN to diagnosis of the skin cancer. Simulations were performed to the Australian Cancer Database (ACD), and results indicated that the suggested technique modified the performance of the method. The method used ANN method that can be considered as an old and less accuracy in these years.

Vocaturo and Zumpano [19] used a method called multi-instance learning (MIL) algorithm to the diagnosis of the melanoma from dysplastic nevi. Simulation results showed that using the MIL technique can be considered as one of the suitable tools for using in skin cancer diagnosis. However, MIL was a simple form of weakly supervised classification technique with sets that can provide weaker results in some cases.

Dey et al. [20] proposed an optimal machine vision technique for the diagnosis of the melanoma. The Bat algorithm was used to improve the accuracy of the diagnosis system. Distance-regularized level-set (DRLS) segmentation method was used for efficient segmentation of the melanoma. The results were then verified by evaluating the important image performance metrics (IPM) on the PH2 database to show the method accuracy.

Literature review showed that although there are different types of diagnosis system for the melanoma detection, numerous research gaps are still to be addressed, for example, higher complexity of some works, complex configuration, and less accuracy. The shortcoming of all research works is given previously after each work. Therefore, this subject is still open and can be developed. However, the main configuration of the Xception network is based on the Inception module [21], blending of the inception modules, convolutional layers, residual connections, and depthwise separable convolutions to improve its efficiency.

The main target of the present research is to deliver a new improved version of Xception based on performing the Swish activation function to diagnose the skin cancer and verify the method by Skin Cancer MNIST: HAM10000 dataset. The presented system classifies the input images into three classes: normal, carcinoma, and melanoma. Furthermore, the results of the proposed Xception architecture are compared with some renowned methods, including VGG16 [22], InceptionV3 [23], AlexNet [24], and the original Xception [25] to show the superiority of the proposed methodology. The configuration of all methods can be achieved by their papers for reproduction. Therefore, the novelty of the presented study is based on proposing an automatic diagnosis method for the skin cancer dermoscopy images based on a new configuration of the XceptionNet. In this study, an improved version of the XceptionNet based on swish activation function has been presented, and the results show a higher accuracy toward the original XceptionNet, and some other related CNN-based methods for skin cancer diagnosis.

## 2. The Modified Xception Network Architecture

The Xception architecture is one of the popular and strong convolutional neural networks that is advanced under different important concepts, like convolutional layer, depth-wise separable convolution layer, residual connections, and inception module [21]. Furthermore, the architecture of CNN for the activation function is essential, wherein *Swish* as a new activation function has been used for developing the traditional activation function [26]. In this study, a Swish activation function has been proposed for improving the Xception based on Swish image classification model for initial melanoma diagnosis [25].

**Figure 1:**
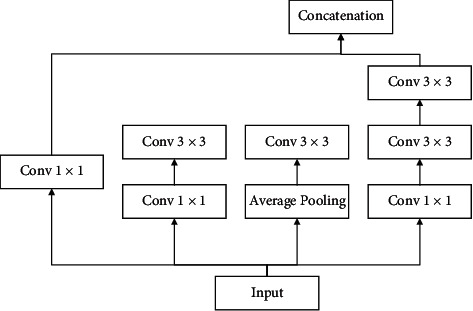
The overall module of an Inception v3.

The Xception is described as a theory based on the Inception module that generates cross-channels correlations and spatial relations within CNN feature maps to be entirely decoupled [27].  Figure 1 shows the overall module of an Inception v3.

**Figure 2:**
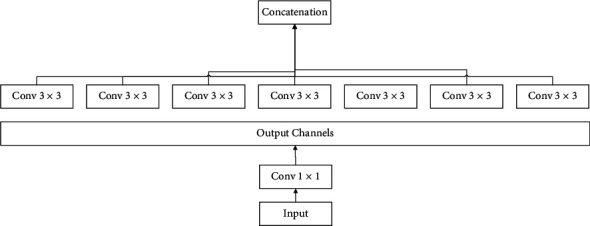
The overall module of an Xception module.

As can be observed from Figure 1, the model is based on cross-channel correlations by input data separation into four ways to convolution size of 1 × 1 and average pooling and mapping correlations by the convolution of size 3 × 3 and finally forwarding to the concatenation layer. The overall module of the studied Xception module has been shown in Figure 2.

As can be seen from Figure 2, in this network, the data from the input uses just one size of 1 × 1 convolution to generate convolution sizes of 3 × 3 with no average pooling, which ensue avoiding overlapping of the output channels to inject to the concatenation. This module is more consistent, stronger, and reliable than the Inception module, which operates correlations of cross-channels and spatial relations with maps fully decoupled. In the following, the stages for the Xception module are explained in detail:

### 2.1. Convolutional Layer

For generating feature maps, the convolution kernels have been separated into input data areas [25]. The different convolution kernels generate the absolute results of the feature maps, such that the position (*i*,  *j*) upon feature value in the feature map as the *k*^th^ layer indicates the *l*^th^, i.e.,

(1) $$\begin{array}{c} S_{i,j,k}^{l}=Bv_{k}^{l}+Wv_{k}^{l}C_{i,j}^{l}, \end{array}$$

where *Wv*_*k*_^*l*^ describes the weight vector, *Bv*_*k*_^*l*^ describes for the bias value of the *k*^th^ filter of the *l*^th^ layer, and *C*_*i*,*j*_^*l*^ describes the input patch center on position (*i*,  *j*) of the *l*^th^ layer.

The *Wv*_*k*_^*l*^ kernel has been generated in sharing the feature map of *S*_*i*,*j*,*k*_^*l*^. This process decreases difficulties and develops the network for graceful model training. Batch normalization is used to insert the convolutional layers of the Xception module, and the activation function is as ReLU, i.e.,

(2) $$\begin{array}{c} \text{RELU}=\left\{\begin{array}{cc} x, & x\geq 0,\\ 0, & x<0, \end{array}\right. \end{array}$$

where *d* describes the input data.

The ReLU activation function is not complicated mathematically with nonlinearity of the network that is vital in convolutional neural network for identifying the nonlinear features, which produce faster convergences and better predictions with less overfitting.

### 2.2. Depthwise Separable Convolution Layer

The depthwise convolutions contain the main part of the Xception modules. These can decrease the computation and the model parameters, which are prepared in depth dimensions and spatial dimensions of color channels. The depthwise convolution makes a filter to the input data set channels of *M* and generates the feature map to define DF × DF × *M*. The depthwise convolution based on the input channel filter is obtained as follows:

(3) $$\begin{array}{c} {\hat{G}}_{k,p,m}=\sum_{i,j,m}{\hat{K}}_{i,j,m}\times F_{k+i-1,p+j-1,m}, \end{array}$$

where $\hat{G}$ describes the alternatives of the feature maps output produced by *F* as the input feature map, and $\hat{K}$ defines the depthwise convolution kernel.

The filter number *m* in $\hat{K}$ is employed to channel the *m*^th^ in *F* for estimation of the output of the feature map. Afterward, the image is presented in multiple channels that can be taken in each color channel. 1 × 1 convolution filters are then used to provide the output to be injected into the next layer. After the depthwise separable convolution layer, batch normalization is utilized, and then using the max-pooling layer, the computational complexity has been decreased.

### 2.3. Residual Connection

For accomplishing the residual connection, the ResNet architecture has been employed, where the internal network performs identity shortcut connections directly into the final layers. By considering the parameters as *p*_*i*_, the residual block is explained as follows:

(4) $$\begin{array}{c} v_{o}=v_{i}+f\left(v_{i},\left[p_{i}\right]\right), \end{array}$$

where *v*_*i*_ and *v*_*o*_ describe the input and the output vectors of the layers, respectively.

**Figure 3:**
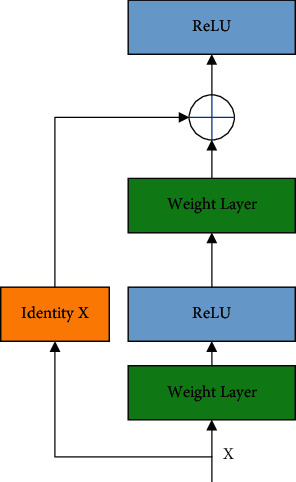
The residual shortcut connection of ResNet.

The benefit of the residual connection is that it avoids signal mitigation by transforming of multiple stacked nonlinearities. It has also quicker training process.  Figure 3 shows the residual shortcut connection of ResNet.

**Figure 4:**
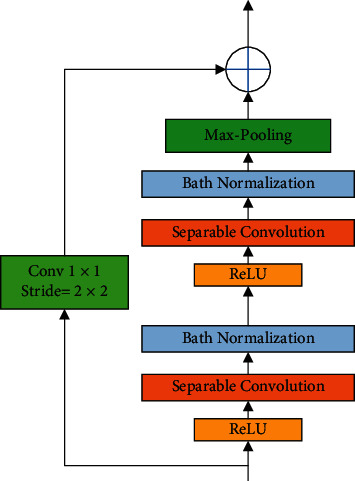
The method of using the residual shortcut connection in Xception.

Also, the method of using the residual shortcut connection in Xception is shown in Figure 4.

As can be observed from Figure 4, the input of *X* can direct a late layer by a shortcut of identity blocks. By considering Figure 4, 1 × 1 convolution operation directed data to a late layer via with a step of 2 × 2. Xception includes a network with 36 convolutional layers that is used for producing the feature extraction. It generates 14 modules that intersperse with residual connections excepting the first and the last modules. In Xception pretrained network, the input image should be of size 299 × 299 × 3.

### 2.4. Swish Activation Function

**Figure 5:**
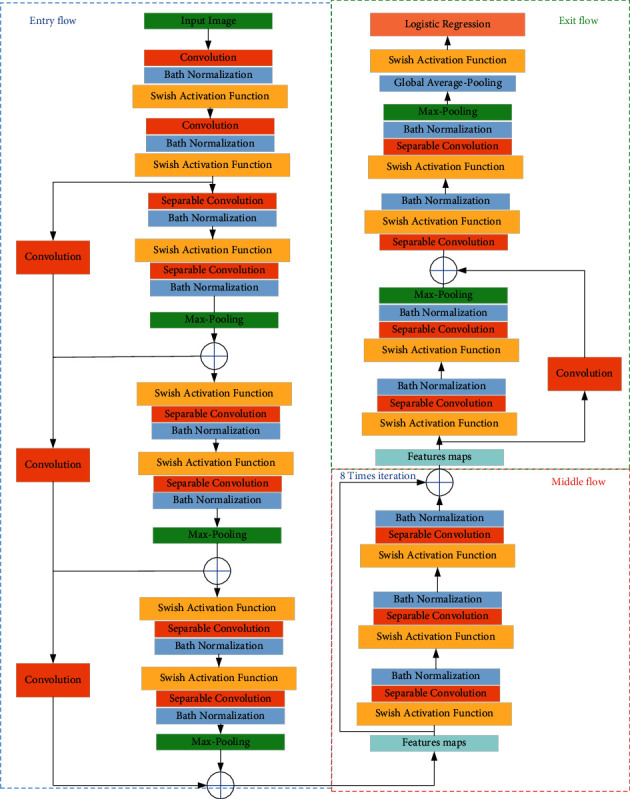
The architecture of the modified Xception network.

Based on a new work from [28], the Swish activation function provides an efficient results for the classification results. In other words, Swish activation function develops the CNN performance rather than the traditional ReLU activation function [25]. The mathematical formulation of the Swish activation function is given as follows:

(5) $$\begin{array}{c} S=m\times \text{sigmoid}\left(\alpha \times m\right), \end{array}$$

where *α* describes an adjustable per-channel parameter, *m* defines the input data, and sigmoid(*α* × *m*) signifies the evaluation of the sigmoid function. The architecture of the modified Xception network is shown in Figure 5.

As can be observed from Figure 5, the modules are similar to the original Xception, and just ReLU function has been replaced with Swish activation function position, which is located before logistic regression and after the global average-pooling.

## 3. Results and Discussion

### 3.1. Dataset

**Figure 6:**
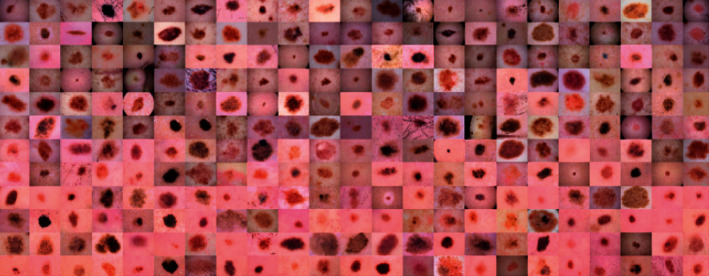
Some examples of the HAM10000 dataset.

The skin cancer benchmark datasets in this study were collected from Skin Cancer MNIST: HAM10000 [29]. This dataset by license number CC BY-NC-SA 4.0 is considered as a guaranteed dataset for the skin cancer diagnosis techniques. The dataset was collected from the Kaggle public Imaging Archive. The dataset includes 10015 JPEG (8-bit color depth) training dermatoscopic images that are collected from different populations collected during a period of 20 years from two different sites, the Department of Dermatology at the Medical University of Vienna, Austria, and the skin cancer practice of Cliff Rosendahl in Queensland, Australia. The Australian site stored images and metadata in PowerPoint files and Excel databases. The Austrian site started to collect images before the era of digital cameras and stored images and metadata in different formats during different time periods. From the literature, different techniques are confirmed based on this dataset [30–33]. In this paper, the data from this benchmark are used to train the proposed Xception network. The data was collected as dermatoscopic images from different populations, acquired and stored by different modalities [34]. 53.3% of lesions were confirmed by histopathology.  Figure 6 shows some examples of the HAM10000 dataset.

We applied the proposed Xception network as a complete diagnosis system for the detection of the skin cancer. Here, we also employed data augmentation. Data augmentation is performed for increasing the number of images for training the CNNs. This is done to compensate the smaller number of training datasets. In other words, Augmentation is utilized to expand the small size datasets by adding supplementary images that are variations of available images in the dataset. This will help improve the ability and performance of the system. There are lots of variations that are introduced for augmentation. In this study, rotation, horizontal shifting, and cropping are used.

One of the transformations in this study is horizontal shift augmentation. A horizontal shift augmentation shifts image pixels horizontally with keeping the image dimension unchanged. This process has floating-point value between 0 and 1 that shows the step size of moving the process. Here, we used 0.3 step size. Another transformation is rotation. During rotation, a rotation angle is specified of specific angle that we want the image to be rotated. This study uses [15, 30, 45, 60] rather than letting it randomly pick it from −90 to 90. Another method for augmentation in this study is based on cropping. Based on cropping in this study, a section (here, center of image) is sampled from the original image and then, it resized to the original image size.

Indeed, the reason of data augmentation here is to increase the quantity of data by adding somewhat altered copies of already existing data or newly created synthetic data from the present data. In other words, we used data augmentation (i.e., shifting, rotation, and cropping) to regularize and help decrease overfitting of data when training the proposed Xception model.

### 3.2. Training and Configuration of the Proposed Xception Network

The dataset has been divided into two groups: 80% for training (8012 images) and 20% (2003 images) for test. In the training procedure, all of the images have been resized to 227 × 227. The CNN model runs 15 times independently to perform the training the dataset; in other words, the proposed network has been performed 15 times in MATLAB environment, and the average results of the model are considered as the measurement values of the model. The simulations were performed on a Core i7 CPU 2.00 GHz laptop, with 2.5 GHz, 16 GB RAM, and 64-bit operating system. The implementation was programmed on MATLAB 2019b as the main programming language on a Windows operating system.  Table 1 indicates the specifications of the hardware and the software.

Also, the model configuration for the prosed CNN contains 12 batch sizes with 2*e* − 2 initial learning rate based on stochastic gradient descent with momentum (SGDM) optimizer. The data configuration is achieved based on trials and errors and close to the [35].

### 3.3. Evaluation Criteria

In this study, four evaluation criteria are utilized to indicate the capability of the proposed system. The mathematical formulation of the utilized measures is briefly given as follows:

(6) $$\begin{array}{c} \text{accuracy}=\frac{\text{TP}+\text{TN}}{\text{TP}+\text{TN}+\text{FP}+\text{FN}},\\ \text{precision}=\frac{\text{TP}}{\text{TP}+\text{FP}}, \end{array}$$

where the accuracy describes the measurements closeness to a specific value, while precision is the measurements closeness to each other and

(7) $$\begin{array}{c} \text{sensitivity}=\frac{\text{TP}}{\text{TP}+\text{FN}},\\ F1‐\text{score}=\frac{2\times \text{precision}\times \text{sensitivity}}{\text{precision}+\text{sensitivity}}, \end{array}$$

where sensitivity (True Positive rate) defines the positives proportion that is correctly recognized. Also, *F*1-score has been achieved by using the precision and sensitivity of the test; i.e., the *F*1-score determines the harmonic mean of the precision and the sensitivity.

In the above equations,

(8) $$\begin{array}{c} \text{TP}:\text{True Positive,}\\ \text{TN}:\text{True Negative,}\\ \text{FP}:\text{False Positive,}\\ \text{FN}:\text{False Negative.} \end{array}$$

### 3.4. Results

In this section, we investigate the method based on some different measurement indicators.  Table 2 reports the performance analysis of the proposed Xception method compared with other studied algorithms.

As can be observed from the results reported in Table 2, the proposed Xception method for the studied dataset offers the highest accuracy rate, which is 100%, and the original Xception, AlexNet, InceptionV3, and VGG16 have been ranked in the next places. The results also show that the sensitivity of the proposed Xception method with 94.05% provides the uppermost toward the others. This shows that how the proposed Xception is good in the test at detecting a positive Melanoma. The results also indicate that the proposed Xception method with 97.07% precision has the highest value, which shows its higher reliability toward the other studied methods. Finally, the *F*1-score of the suggested technique is 0.9553, which is the highest value among the others. In *F*1-score indicator, if the value gets closer to 1, it has the maximum precision and sensitivity.

**Figure 7:**
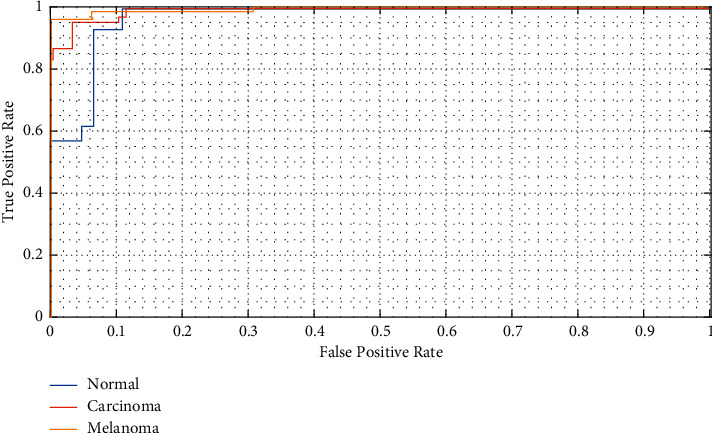
The Receiver Operating Characteristics curve for three classes including melanoma, carcinoma, and normal.

For more declaration, the Receiver Operating Characteristics (ROC) curve for three classes, i.e., melanoma, carcinoma (BCC), and Normal, is shown in Figure 7. The ROC curve is a graphical profile that indicates the diagnostic ability of a binary classifier system as its discrimination threshold is varied. The method signifies the diagnosis ability for the model with measuring the separability degree among different classes. More area under ROC shows better results for the model; i.e., the ROC area will be ideal if the area under the diagram is 1, and it will be poor if the area is 0.

As can be observed from Figure 7, the average area under this curve for melanoma class is 1.0, normal class is 0.98, and pneumonia class is 0.98. The main cause of that the area of classes normal and pneumonia is 0.98, as our model has forecasted 3 false positives in case of pneumonia and 1 false negative for normal patients, while melanoma is 1 due to the absence of no false negatives and false positives.

**Figure 8:**
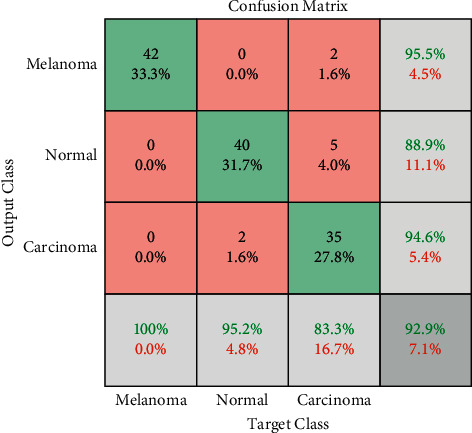
The confusion matrix of true class and predicted class for the traditional Xception [25].

**Figure 9:**
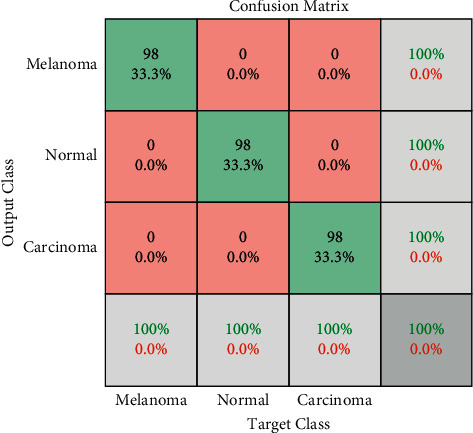
The confusion matrix of true class and predicted class for the proposed Xception.

Based on the results, it is clear that both the proposed and the original Xception models provide the classification performance probability for three classes to progress an image diagnosis for the melanoma screening. The classification accuracy of the suggested Xception is better than the original Xception model as determined in the results. In defining the performance of the model classification, a confusion matrix of true class and predicted class for Xception has been shown in Figure 8 [25] and the confusion matrix of the proposed Xception method is shown in Figure 9.

As can be observed from the confusion matrix results for the diagnosis of the melanoma based on the original and the proposed Xception models for the diagnosis of the three-class dataset, the proposed method provides a high accuracy. Indeed, the original Xception achieved true prediction of melanoma in 42 images, accounting for 33.30%, true prediction of normal cases in 40 images, or 31.7%, with false prediction of 68.3%; and true prediction of the pneumonia is 27.8% of the accuracy.

Also, the proposed Xception model achieved true prediction of melanoma in 98 images, accounting for 33.33%, true prediction of normal cases in 40 images, or 33.33%, and true prediction of the pneumonia is 33.33% of the accuracy. Thus, the proposed Xception model based on Swish activation function provides higher accuracy compared with original Xception model.

As can be observed from the results, the proposed method has better effectiveness for the skin cancer diagnosis. However, there are some cases that ca be more improved for resolving its limitations: the method needs a large amount of data to deliver better results. Due to the need for the complex data, its training is expensive; i.e., it needs expensive GPU for better performance. Selecting a good topology and its other parameters is hard, which can be even harder for the less skilled people.

## 4. Conclusions

Among different types of cancer, skin cancer is considered as one of the most widely distributed ones. Melanoma is one of the most dangerous forms of skin cancer. If this type of cancer is diagnosed early, it can be treated 100%. But if it becomes aggressive and spreads to other tissues in the body, it will not be possible to treat it. Therefore, early detection of melanoma can increase a person's chances of recovery and prevention of transmission to others. This study proposed a new architecture of Xception deep network as a convolutional neural network to provide an efficient diagnosis system for melanoma detection. Two main improvements of this model are to use Swish activation function and depthwise separable convolutions to improve the accuracy of the classification stage of the CNN. The proposed Xception method was then implemented to MNIST skin cancer dataset, and the results were compared with some state-of-the-art methods. Results showed that the proposed method, with 100% accuracy, 94.05% sensitivity, 97.07% precision, and 95.53% *F*1-score, provided the highest performance among the others.

## Figures and Tables

**Table 1:** The specifications of the hardware and the software.

Name	Setting
Hardware	Intel® Core™ i7-4720HQ
CPU	1.60 GHz
RAM	16 GB
Frequency	1.99 GHz
Operating system	Windows 10
Programming software	MATLAB R2019b

**Table 2:** The performance analysis of the proposed Xception method compared with other studied algorithms.

Method	Accuracy (%)	Sensitivity (%)	Precision (%)	*F*1-score
VGG16 [22]	48.99	53.7	46.97	50.11
InceptionV3 [23]	52.99	53.99	52.99	53.48
AlexNet [24]	75.99	76.99	75.99	76.48
Xception [25]	92.90	91.99	91.99	91.99
Proposed Xception	100	94.05	97.07	95.53

## Data Availability

The database utilized in this paper can be downloaded from https://www.kaggle.com/kmader/skin-cancer-mnist-ham10000.

## References

[1] Adegun A., Viriri S. (2021). Deep learning techniques for skin lesion analysis and melanoma cancer detection: a survey of state-of-the-art. *Artificial Intelligence Review*.

[2] Javaid A., Sadiq M., Akram F. Skin cancer classification using image processing and machine learning.

[3] Jiang S., Li H., Jin Z. (2021). A visually interpretable deep learning framework for histopathological Image-based skin cancer diagnosis. *IEEE Journal of Biomedical and Health Informatics*.

[4] Reinaldo F. P., Vishnevski M. (2020). Computer-Aided Diagnosis of Skin Cancer: A Review. *Current Medical Imaging*.

[5] Clinton S. K., Giovannucci E. L., Hursting S. D. (2020). The World Cancer Research Fund/American Institute for Cancer Research third expert report on diet, nutrition, physical activity, and cancer: impact and future directions. *Journal of Nutrition*.

[6] Xu Z., Sheykhahmad F. R., Ghadimi N., Razmjooy N. (2020). Computer-aided diagnosis of skin cancer based on soft computing techniques. *Open Medicine*.

[7] Khan M. Q., Hussain A., Rehman S. U. (2019). Classification of melanoma and nevus in digital images for diagnosis of skin cancer. *IEEE Access*.

[8] Bharathi S., Premchand J., Nivedeethaa A., Kousiya M., Ajay Kumar V. (2021). Identification of melanoma from nevus images. *Journal of Physics: Conference Series*.

[9] Rokhana R., Herulambang W., Indraswari R. Deep convolutional neural network for melanoma image classification.

[10] Costa A., Kieffer Y., Scholer-Dahirel A. (2018). Fibroblast heterogeneity and immunosuppressive environment in human breast cancer. *Cancer Cell*.

[11] Babar M., Butt R. T., Batool H., Asghar M. A., Majeed A. R., Khan M. J. A refined approach for classification and detection of melanoma skin cancer using deep neural network.

[12] Brinker T. J., Schmitt M., Krieghoff-Henning E. I. (2021). Diagnostic performance of artificial intelligence for histologic melanoma recognition compared to 18 international expert pathologists. *Journal of the American Academy of Dermatology*.

[13] Wang Q., Sun L., Wang Y. (2020). Identification of melanoma from hyperspectral pathology image using 3D convolutional networks. *IEEE Transactions on Medical Imaging*.

[14] Modi H., Chhabra B., Mahalakshmi P. (2021). Melanoma Classification: A Survey. *Annals of the Romanian Society for Cell Biology*.

[15] Azad M. M., Ganapathy A., Vadlamudi S., Paruchuri H. (2021). Medical diagnosis using deep learning techniques: a research survey. *Annals of the Romanian Society for Cell Biology*.

[16] Pérez E., Reyes O., Ventura S. (2021). Convolutional neural networks for the automatic diagnosis of melanoma: an extensive experimental study. *Medical Image Analysis*.

[17] Bansal P., Kumar S., Srivastava R., Agarwal S. (2021). Using transfer learning and hierarchical classifier to diagnose melanoma from dermoscopic images. *International Journal of Healthcare Information Systems and Informatics*.

[18] Razmjooy N., Sheykhahmad F. R., Ghadimi N. (2018). A hybrid neural network – world Cup optimization algorithm for melanoma detection. *Open Medicine*.

[19] Vocaturo E., Zumpano E. Dangerousness of dysplastic nevi: a multiple instance learning solution for early diagnosis.

[20] Dey N., Rajinikanth V., Lin H., Shi F. (2021). A study on the bat algorithm technique to evaluate the skin melanoma images. *Applications of Bat Algorithm and its Variants*.

[21] Chollet F. Xception: deep learning with depthwise separable convolutions.

[22] Manasa K., Murthy D. G. V. (2021). Skin cancer detection using VGG-16. *European Journal of Molecular & Clinical Medicine*.

[23] Ridell P., Spett H. (2017). *Training Set Size for Skin Cancer Classification Using Google’s Inception V3*.

[24] Hosny K. M., Kassem M. A., Foaud M. M. (2019). Classification of skin lesions using transfer learning and augmentation with Alex-net. *PloS one*.

[25] Gavrilov D., Lazarenko L., Zakirov E. AI recognition in skin pathologies detection.

[26] Kassani S. H., Kassani P. H., Wesolowski M. J., Schneider K. A., Deters R. Breast cancer diagnosis with transfer learning and global pooling.

[27] Kuritcyn P., Benz M., Dexl J., Bruns V., Hartmann A., Geppert C. Comparison of CNN models on a multi-scanner database in colon cancer histology.

[28] Hussain E., Hasan M., Rahman M. A., Lee I., Tamanna T., Parvez M. Z. (2021). CoroDet: a deep learning based classification for COVID-19 detection using chest X-ray images. *Chaos, Solitons & Fractals*.

[29] Codella N., Rotemberg V., Tschandl P. (2019). Skin lesion analysis toward melanoma detection 2018: a challenge hosted by the international skin imaging collaboration (isic). https://arxiv.org/abs/1902.03368.

[30] Nugroho A. A., Slamet I., Sugiyanto (2019). Skins cancer identification system of HAMl0000 skin cancer dataset using convolutional neural network. *AIP Conference Proceedings*.

[31] Chaturvedi S. S., Gupta K., Prasad P. S. Skin lesion analyser: an efficient seven-way multi-class skin cancer classification using mobilenet.

[32] Garg R., Maheshwari S., Shukla A. (2021). Decision support system for detection and classification of skin cancer using CNN. *Innovations in Computational Intelligence and Computer Vision*.

[33] Le D. N., Le H. X., Ngo L. T., Ngo H. T. (2020). Transfer learning with class-weighted and focal loss function for automatic skin cancer classification. https://arxiv.org/abs/2009.05977.

[34] Tschandl P., Rosendahl C., Kittler H. (2018). The HAM10000 dataset, a large collection of multi-source dermatoscopic images of common pigmented skin lesions. *Scientific Data*.

[35] Singh K. K., Siddhartha M., Singh A. (2020). Diagnosis of coronavirus disease (covid-19) from chest x-ray images using modified xceptionnet. *Romanian Journal of Information Science and Technology*.

